# The role of lipid metabolism imbalance in copper-induced PANoptosis in broiler kidney

**DOI:** 10.1016/j.psj.2025.105549

**Published:** 2025-07-15

**Authors:** Feiyang Ma, Yihui Huo, Jianzhao Liao, Guangqing Xu, Zhekai Wang, Shaojun He

**Affiliations:** a College of Animal Science, Anhui Science and Technology University, Chuzhou, Anhui 233100, PR China; b College of Veterinary Medicine, South China Agricultural University, Guangzhou, Guangdong 510642, PR China; c College of Veterinary Medicine, China Agricultural University, Beijing 100091, PR China

**Keywords:** Copper, Lipid metabolism, PANoptosis, Kidney, Broiler

## Abstract

Copper (Cu) is widely used in both agriculture and industry and may pose toxic risks to animals and public safety if overused. In order to gain a more profound insight into the nephrotoxic effects of Cu, a detailed analysis was performed of its impact on renal PANoptosis, with particular attention being paid to the possible involvement of lipid metabolism disorders in the kidney. In this study, one-day-old chicks were fed diets with varying Cu levels (11, 110, 220, and 330 mg/kg) over a period of 49 days. Our findings indicated that excessive Cu exposure led to vacuolar degeneration, fibrosis and mitochondrial damage in the kidney. Moreover, the assay results demonstrated that elevated Cu levels led to disturbances in lipid synthesis and catabolism, as well as the activation of lipophagy in broiler kidneys. Concurrently, genetic and protein analysis demonstrated that excess Cu triggered pyroptosis (IL-18, NLRP3, GSDMD, Caspase-1), necrosis (MLKL, Caspase-7, Caspase-8) and apoptosis (Bcl-2, Cleaved-Caspase-9/Caspase-9, Cleaved-Caspase-9/Caspase-9), ultimately resulting in PANoptosis in the chicken kidney. Furthermore, the bioinformatics analysis indicated a correlation between lipid metabolism and PANoptosis-related markers. The aforementioned results indicate that Cu-induced disruption to lipid metabolism may contribute to the process of PANoptosis in broiler kidneys.

## Introduction

Copper (Cu), an essential trace element in the body, is carefully regulated at both tissue and cellular levels to maintain appropriate uptake, distribution, utilization, and elimination. Moreover, as a catalytic cofactor in the field of redox chemistry, Cu may be implicated in the modulation of diverse enzyme activities. The extensive use of high concentrations of Cu in feed additives, fertilizers, and pesticides has led to considerable environmental contamination (Zhen, et al., 2022). With the rapid advancement of agriculture and industry, Cu pollution has emerged as a global issue (Chen, et al., 2022; Setu and Strezov, 2025). Research indicates that elevated Cu levels can cause kidney damage, metabolic disturbances, and even organ failure (Casanova, et al., 2024; Qiao, et al., 2021; Rosa, et al., 2021). Meanwhile, excess Cu can lead to impairment of mitochondrial function, resulting in cell death (Xue, et al., 2023). Relevant research has shown that excess Cu modulates altered levels of renal mitophagy, and apoptosis (Li, et al., 2023; Liao, et al., 2020). Nevertheless, the in-depth mechanism by which high Cu exposure induces nephrotoxicity is unclear.

Fatty acids (FAs) are essential for energy metabolism, serving as the primary means of energy storage and circulation. FA metabolism consists of the uptake, synthesis, and breakdown of fatty acids, processes that are interdependent and closely regulated. Among them, mitochondria are an important site for FA β-oxidation catabolism, which produces adenosine triphosphate (ATP) to provide energy for the cell (Lin, et al., 2024). Lipid metabolism disorders contribute to the onset of various diseases, including metabolic syndrome, obesity, and type 2 diabetes. Meanwhile, studies have shown a complex relationship between Cu and FA metabolism. ATPase Cu transporting beta (ATP7B) is pivotal in regulating intracellular Cu transport and metabolism. Loss of ATP7B function leads to hepatic Cu overload, which can induce downregulation of genes related to lipid metabolism (Seessle, et al., 2011). Meanwhile, it was investigated that Cu exposure induces hepatic oxidative stress, promotes lipogenesis and inhibits fat oxidation, which can induce hepatic lipid deposition (Zhong, et al., 2022).

Programmed cell death (PCD) includes pyroptosis, apoptosis, and necroptosis, each initiated, transmitted, and executed by intricate molecular mechanisms. These pathways engage in extensive crosstalk, leading to a newly recognized form of cell death that integrates elements of all three, termed PANoptosis (Pandeya and Kanneganti, 2024). The process of PANoptosis is subject to regulation by upstream receptors and molecular signals, which combine to form a polymeric structure known as the PANoptosome. Meanwhile, the PANoptosome functions as a molecular scaffold, thereby enabling the coupling and binding of key molecules implicated in pyroptosis, apoptosis, and necroptosis (Gao, et al., 2024). It has been shown that oxidative stress in mitochondria induces reactive oxygen species (ROS) production and cytochrome C release (Cyt C), thereby inducing PANoptosis (Liu, et al., 2023). Recent studies have indicated that heavy metals can lead to renal injury and mitochondrial dysfunction (Cong, et al., 2025; Sanders, et al., 2019). Nevertheless, it remains unclear whether high Cu levels can induce PANoptosis in chicken kidneys.

Although previous studies have shown that high Cu induces mitochondrial damage in broiler kidneys (Liao, et al., 2021; Liao, et al., 2020), whereas the effects on lipid metabolism and PANoptosis and in broiler kidneys are not yet clear. This study aimed to investigate the effects of Cu on renal lipid metabolism and its role in Cu-induced PANoptosis in broiler, with the goal of elucidating mechanisms of Cu-induced kidney damage and providing insights for preventing and treating nephrotoxicity from environmental toxicants.

## Materials and methods

### Animals and treatment

This animal study received approval from the Ethics Committee of South China Agricultural University. A total of 240 healthy one-day-old white feather broilers were randomly divided into four groups: control group (Control), group receiving 110 mg/kg Cu (Group I), group receiving 220 mg/kg Cu (Group Ⅱ), and group receiving 330 mg/kg Cu (Group Ⅲ) (Yang, et al., 2021; Zhong, et al., 2023). CuSO4, an analytical reagent with a purity of 99.0 %, purchased from Macklin (China), was utilized as the source of Cu. At the conclusion of week 7, a random selection of ten animals from each group was euthanized under ether anesthesia. Serum and kidney tissue samples were then collected. Each kidney sample was subjected to partial fixation with paraformaldehyde and glutaraldehyde, followed by partial freezing and storage at -80°C.

### Serum biochemical detection

Following the collection of blood samples from broilers in each group, the serum was separated, and the levels of creatinine (Crea) and blood urea nitrogen (BUN) were assessed using a Myriad BS-380 automatic biochemical analyzer (Myriad Biomedical Electronics Co., Ltd., Shenzhen).

### Copper content detection

Kidney treatments for the broilers were conducted in accordance with previously established methods (Ma, et al., 2023). Accurately weigh 2 mL of serum and 0.10 mg of kidney samples in 15 mL test tubes respectively and add 5 mL of HCl. Heat to dissolve for 15 min, then add 2.5 mL of HNO_3_ and continue heating until the sample is completely decomposed. The solution should then be dispensed to 10 mL and left to test. Afterwards, the levels of relevant elements were detected by flame atomic absorption.

### Histological observation

The kidney tissues, after fixation in paraformaldehyde, were removed and sectioned into smaller pieces before being embedded using standard procedures (Ma, et al., 2022). These sections underwent hematoxylin and eosin staining to assess cellular structures (Solarbio, China). Furthermore, Masson staining was conducted following the guidelines included with the staining kit (Solarbio, China).

### Ultrastructural observation

The kidney samples were preserved in 2.5 % glutaraldehyde, rinsed in PBS and post-fixed in 1 % osmium tetroxide for a duration of 24 hours. Subsequently, the samples were immersed in a solution of acetone and resin, then mounted in pure resin and cut on an ultramicrotome. Semi-thin sections were prepared and subsequently stained with uranyl acetate prior to observation by means of transmission electron microscopy (TEM).

### Immunohistochemical assay

The kidney sections were initially deparaffinized using xylene and underwent antigen retrieval in citrate buffer before being blocked with 10 % horse serum. Following this, the sections were incubated for 16 hours with antibodies against IL-1 and NLRP3. Staining was performed using diaminobenzidine (DAB) for 2 minutes, followed by an 8-minute counterstaining with hematoxylin. Finally, the sections were sealed and examined under a microscope to evaluate the results (Leica, Germany).

### Immunofluorescence assay

The pre-treatment procedure, similar to standard immunohistochemistry, was followed by a 14-hour incubation with antibodies targeting Caspase-1, Caspase-3, Caspase-8, RIPK3, and MLKL. Afterward, a secondary antibody was applied for 1 hour, and DAPI staining (Beyotime, China) was subsequently performed. The results were then captured using confocal microscopy (Leica, Germany). The results were subjected to analysis using ImageJ software.

### RT-qPCR

An appropriate quantity of renal tissue was treated with Trizol (Takara, Japan) to isolate total RNA, following the previously established protocol (Huo, et al., 2023). Reverse transcription was performed using the ABScript Neo RT Master Mix for qPCR, which includes a gDNA Remover (ABclonal, China). The specific primers utilized in this study are listed in **Table S1**. qRT-PCR was conducted with the Light Cycler480Ⅱ system, employing the BrightCycle Universal SYBR Green qPCR Mix with UDG (ABclonal, China). The relative changes in mRNA levels were subsequently analyzed using the 2^−△△CT^ method.

### Western blot analysis

An appropriate quantity of kidney tissue was homogenized in RIPA buffer supplemented with PMSF (Beyotime, China). The concentration of protein was determined using the BCA Protein Detection Kit (Vazyme, China), adhering to the established protocol (Zhong, et al., 2023). The primary antibodies employed in the analysis are detailed in **Table S2**, followed by incubation with the respective secondary antibodies from CWBIO (1:5000, China). The resulting protein lines were then detected by ECL and analyzed using ImageJ software.

### Correlation analysis

Cluster heatmapping was performed using Heml software. Pearson's correlation coefficients (PCC) were computed with the assistance of Origin Pro software developed by Microcal in the USA (Ma, et al., 2022).

### Statistical analysis

The *t*-test was employed to compare the means of the two groups. Furthermore, one-way analysis of variance (ANOVA) and Dunnett's post hoc test were employed for the purpose of conducting multiple comparisons. The data were presented as mean ± standard error of the mean (SEM). Statistical graphics were generated with GraphPad Prism 8.0 (GraphPad Inc., La Jolla, CA, USA). Significance levels were denoted as follows: * for *P* < 0.05, ** for *P* < 0.01, and *** for *P* < 0.001.

## Results

### Effect of Cu on kidney damage

**Fig. 1:**
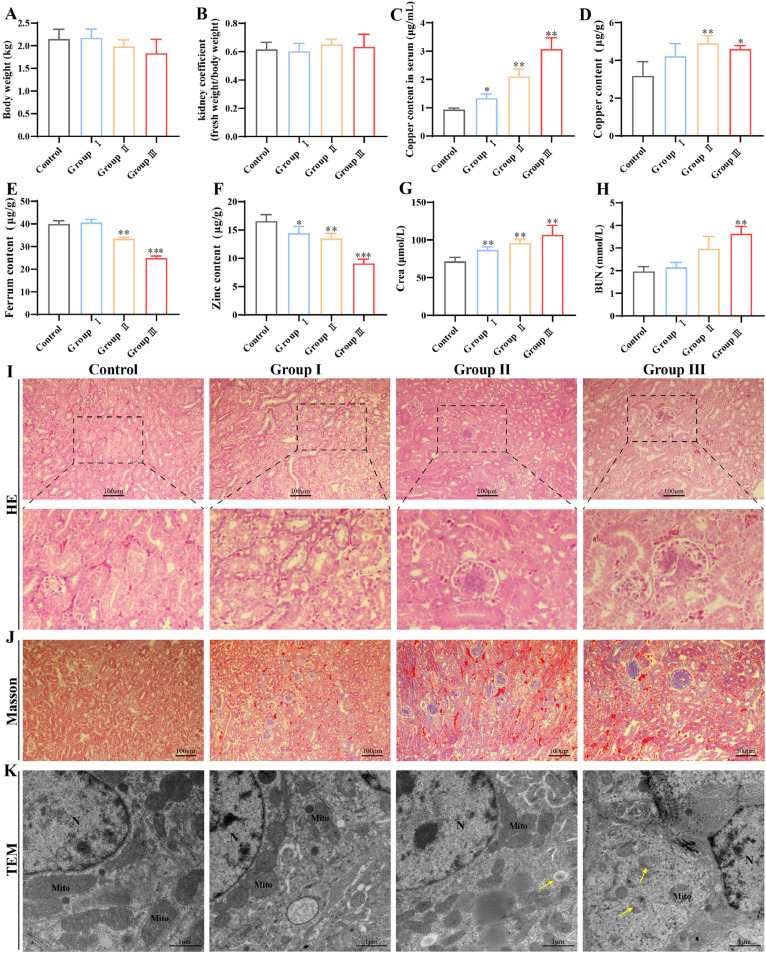
Effects of Cu exposure on histological morphology in kidney. (A) Chicken body weight at 7 weeks. (B) Chicken kidney coefficient. (C) Cu content in chicken serum. (D) Cu content in chicken kidney. (E) Fe content in chicken kidney. (F) Zn content in chicken kidney. (G) Crea content in chicken serum. (H) BUN content in chicken serum. (I) HE staining results. (J) Results of Massoon staining. (K) The ultrastructural observation. The yellow arrow denotes the location of damaged mitochondria. “N” means cell nucleus. “Mito” means mitochondria. All date were expressed as mean ± SEM; n=6 for each group. “*” indicates statistically significant with the control group (*P<0.05, **P<0.01, ***P<0.001). The same as follows.

The analysis of Cu's impact on broilers and their kidneys revealed a tendency for reduced body weight in broilers from groups Ⅱ and Ⅲ than control group (Fig. 1**A**). However, no significant changes were detected in kidney coefficients (Fig. 1**B**). The serum Cu assay results of the broilers in the excess Cu-treated groups demonstrated a notable upregulation (*P* < 0.05) of both then the control group (Fig. 1**C**). Conversely, the renal Cu assay results demonstrated no significant alteration in group I and a notable elevation (*P* < 0.05) in groups Ⅱ and Ⅲ in comparison to the control group (Fig. 1**D**). Furthermore, the results of the assays for zinc and iron content demonstrated a substantial decline (*P* < 0.05) in the group with excess Cu (Fig. 1**E-F**). These findings indicate that Cu excess may influence the growth and development of broilers, potentially disrupting the homeostasis of trace elements in serum and kidneys.

Serological tests showed that Crea and BUN levels were significantly up-regulated (*P* < 0.05) with positive correlation with Cu dose (Fig. 1**G-H**). Additionally, renal injury could be assessed by pathological and ultrastructural observations. As illustrated in Fig. 1**I**, the degeneration of renal tubular epithelial cells was evident in all treatment groups, with a particularly pronounced effect observed in group III, as indicated by the red arrows. In parallel, Masson staining demonstrated a notable elevation in renal fibrosis in response to Cu overload, with a particularly pronounced increase observed in group Ⅲ (Fig. 1**J**). Projected electron microscopy results similarly demonstrated that elevated Cu levels can result in mitochondrial cristae damage and vacuolization (Fig. 1**K**).

### Effects of Cu on exposure lipid metabolism and lipophagy in kidney

**Fig. 2:**
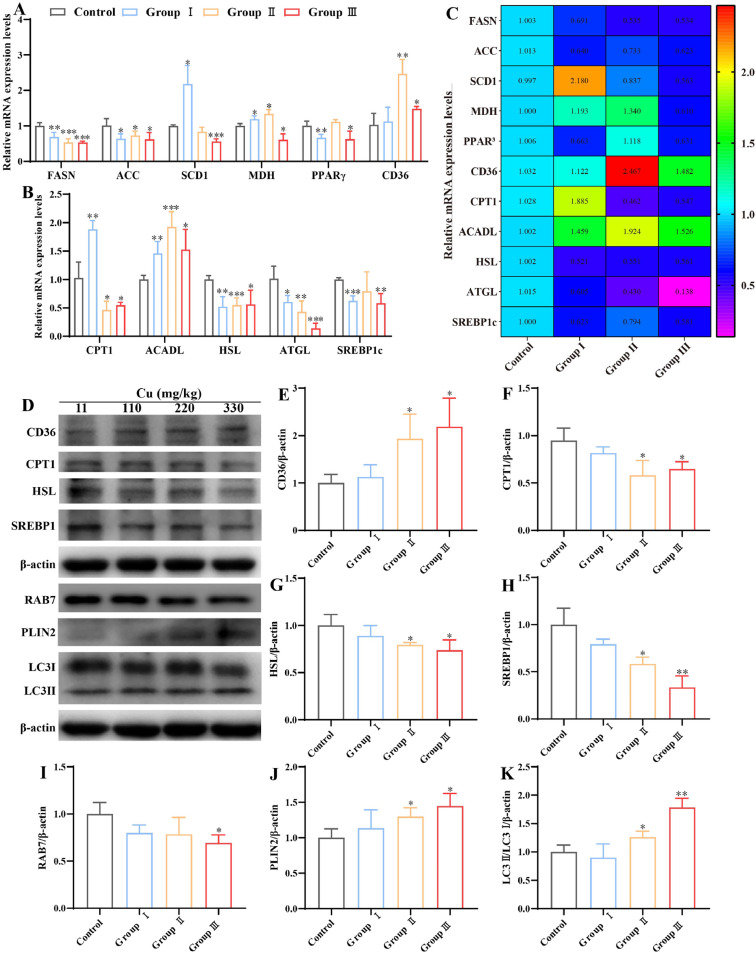
Effects of Cu exposure on lipid metabolism and lipophagy in kidney. (A-B) mRNA expression level of lipid metabolism and lipophagy. (C) Heat map. (D) Western blot result. (E-K) Quantitative analysis of protein expressions related to lipid metabolism and lipophagy.

The present study investigates the potential effects of excess Cu on renal lipid metabolism in broilers, we study examined changes in lipid metabolism-related indices. As illustrated in Fig. 2**A-B**, the genes level of FASN, ACC, HSL, and ATGL were significantly reduced in high Cu groups compared to the control group (*P* < 0.05). In contrast, PPARγ and SREBP1c genes was significantly lowered in groups I and Ⅲ compared to the control group (*P* < 0.05). Furthermore, a significant upregulation of ACADL gene levels was observed in the high Cu groups (*P* < 0.05). CD36 gene levels were found to be significantly increased in groups Ⅱ and Ⅲ (*P* < 0.05). Levels of the SCD1 gene were found to be significantly increased in group I and significantly decreased in group Ⅲ in comparison to the control group (*P* < 0.05). Whereas MDH gene was significantly upregulated in group I and Ⅱ and significantly downregulated in group Ⅲ (*P* < 0.05). In addition, a significant upregulation of CPT1 gene levels was observed in group I, while a significant downregulation was evident in groups Ⅱ and Ⅲ, in comparison to the control group (*P* < 0.05). Additionally, lipid metabolism and lipophagy-related proteins are depicted in Fig. 2**D**. The protein levels of CD36, PLIN2, and the ratio of LC3Ⅱ to LC3Ⅰ were significantly higher in groups Ⅱ and Ⅲ (*P* < 0.05) (Fig. 2**E, J, K**). Similarly, the proteins CPT1, HSL, SREBP1, and RAB7 also showed decreased levels in groups Ⅱ and Ⅲ than control group (*P* < 0.05) (Fig. 2**F-I**). These findings indicate that excessive Cu disrupts lipid metabolism and lipophagy in the kidneys.

### Effects of Cu exposure on pyroptosis in kidney

**Fig. 3:**
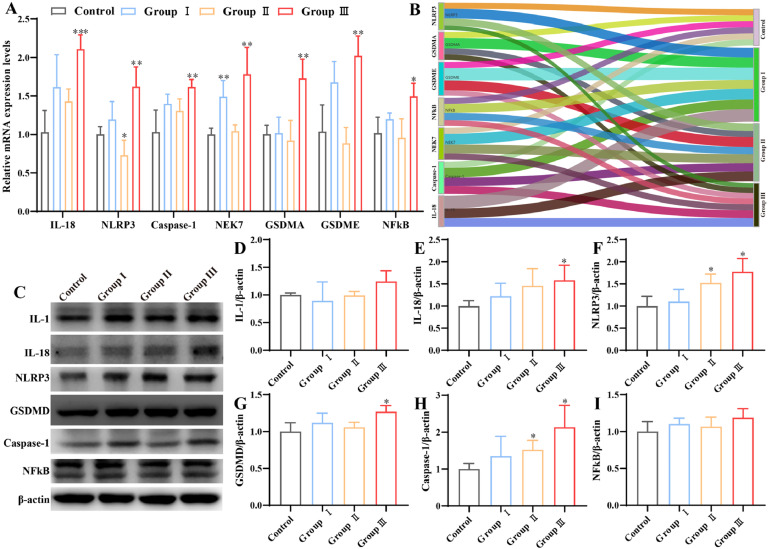
Effects of Cu exposure on pyroptosis in chicken kidney. (A) mRNA expression of mitochondrial dynamics protein. (B) Heat map. (C) Western blot result. (D-I) Results of quantitative Western blot analysis of pyroptosis-associated proteins.

**Fig. 4:**
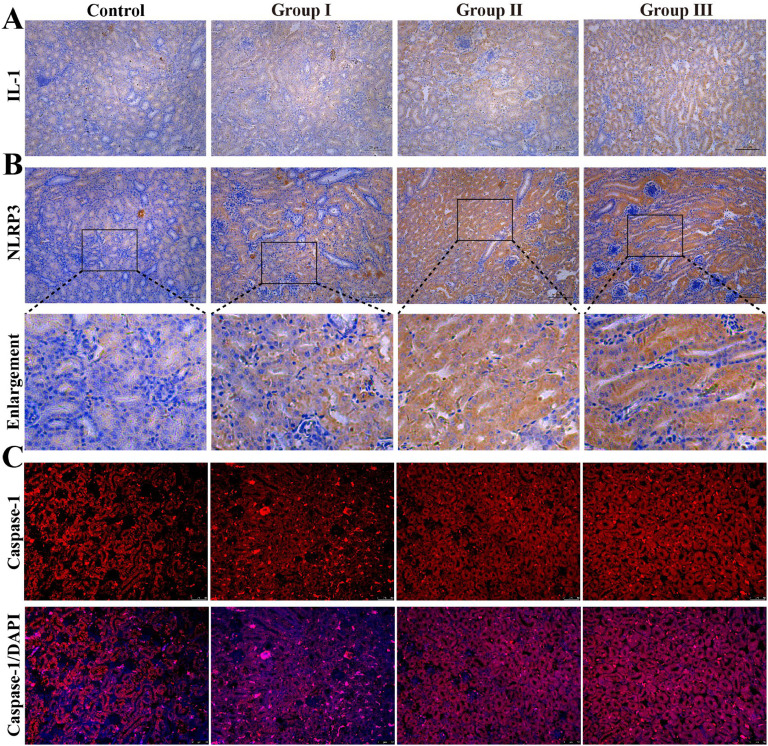
Immunohistochemical and immunofluorescence analyses in chicken kidney. (A-B) Immunohistochemistry staining analyzes the expression of IL-1 and NLRP3. (C) Immunofluorescence staining was used to analyze the expression of Caspase-1.

The present study assessed changes in the levels of pyroptosis-related indices. At the gene level, results revealed that NEK7 levels were significantly elevated in group I (*P* < 0.05), while NLRP3 levels were notably decreased in group Ⅱ compared to the control group (*P* < 0.05) (Fig. 3**A**). In addition, the mRNA levels of IL-18, NLRP3, Caspase-1, NEK7, GSDMA, GSDME, and NFkB were observed to be elevated in group Ⅲ (*P* < 0.05). Fig. 3**C** illustrates the proteins involved in the pyroptosis pathway. The protein levels of IL-18, NLRP3, GSDMD, and Caspase-1 were elevated in group Ⅲ. However, the levels of IL-1 and NFkB did not demonstrate a statistically significant change in the high Cu groups when compared to the control group (Fig. 3**D-I**). The immunohistochemical results demonstrated a gradual increase in the number of IL-1 and NLRP3 protein-positive particles with an increase in Cu concentration (Fig. 4**A-B**). Similarly, immunofluorescence results showed that Caspase-1 fluorescence intensity increased with higher concentrations of Cu (Fig. 4**C**). The aforementioned results indicated that elevated Cu levels resulted in the induction of pyroptosis in broiler kidneys. These results indicated that elevated Cu levels resulted in the activation of pyroptosis in broiler kidneys.

### Effects of Cu exposure on mitochondria-mediated apoptosis in kidney

**Fig. 5:**
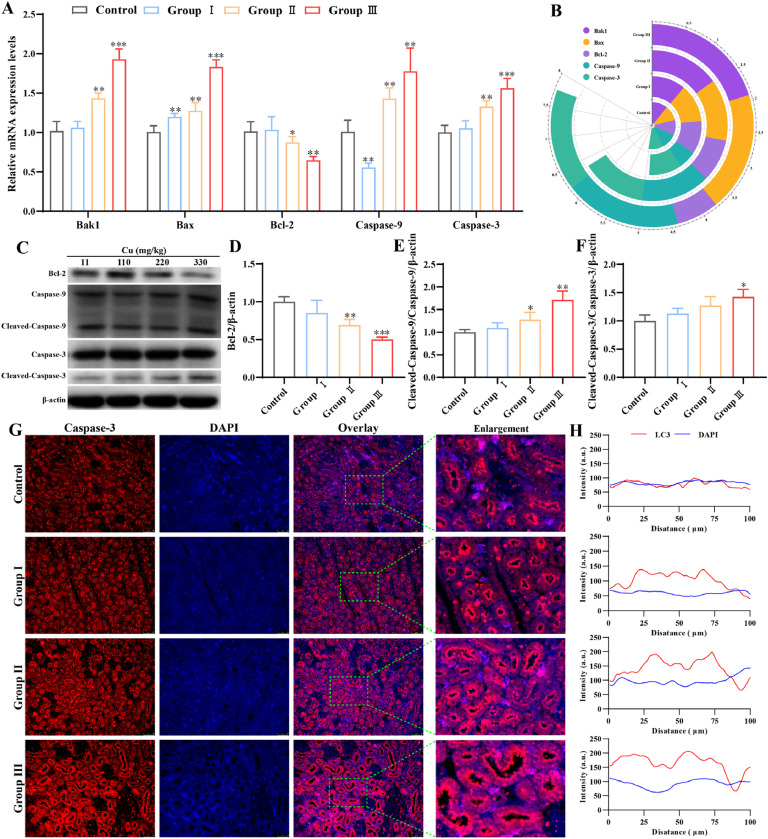
Effects of Cu exposure on apoptosis in chicken kidney. (A) Relative mRNA expression of apoptosis genes in kidney. (B) Heat map. (C) Western blot result of apoptosis-associated proteins. (D-F) Results of quantitative Western blot analysis of apoptosis-associated proteins. (G) Immunofluorescence staining was used to analyze the expression of Caspase-3 in chicken kidney. (H) Fluorescence intensity analysis.

To ascertain whether Cu toxicity can instigate mitochondrial apoptosis, an analysis was conducted to determine the alterations in mitochondrial apoptosis-related biomarkers. As illustrated in Fig. 5**A**, the gene-level results revealed a significant elevation in the levels of Bak1 (BCL2-antagonist/killer 1), Bax (BCL-2-associated X protein), Caspase-9 and Caspase-3 in groups Ⅱ and Ⅲ (*P* < 0.05), while the levels of Bcl-2 (B-cell lymphoma-2) exhibited a marked reduction in groups Ⅱ and Ⅲ in contrast to the control group (*P* < 0.05). As in Fig. 5**C**, an additional examination was conducted to investigate the associated protein levels. Protein analyses showed that Bcl-2 protein results were markedly diminished in group Ⅱ and Ⅲ (*P* < 0.05) (Fig. 5**D**), and Cleaved-Caspase-9/Caspase-9 and Cleaved-Caspase-3/Caspase-3 were found to be elevated in group Ⅱ and Ⅲ than the control group (*P* < 0.05) (Fig. 5**E-F**). Similarly, the immunofluorescence analysis revealed that elevated Cu levels resulted in an increase in the fluorescence intensity of the Caspase-3 protein (Fig. 5**G-H**). These results indicated that an excess of Cu trigger mitochondria-mediated apoptosis in the kidneys of chickens.

### Effects of Cu exposure on necroptosis in kidney

**Fig. 6:**
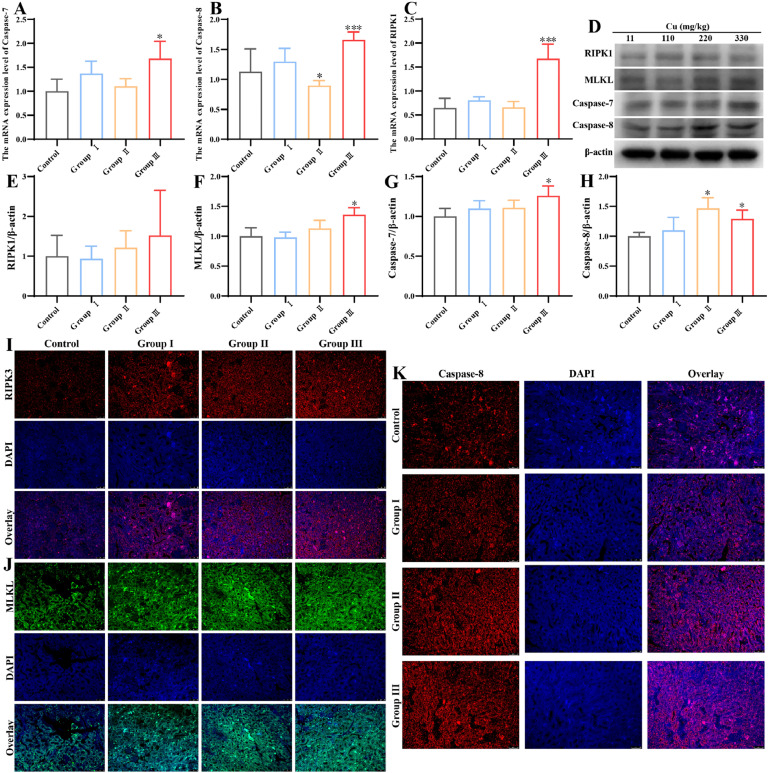
Effects of Cu exposure on necrosis in chicken kidney. (A-C) Relative mRNA expression of necrosis genes in kidney. (D) Western blot result of necrosis-associated proteins. (E-H) Results of quantitative Western blot analysis of necrosis-associated proteins. (I-K) Immunofluorescence staining was employed for the analysis of RIPK3, MLKL, and Caspase-8 expression in chicken kidney.

The present study provided evidence that high Cu levels resulted in a notable elevation in Caspase-7, Caspase-8 and RIPK1 (receptor-interacting protein kinase 1) gene expression in group Ⅲ, when contrasted with the control group (*P* < 0.05) (Fig. 6**A-C**). The results of the protein level detection are presented in Fig. 6**D**. The data showed that the protein levels of MLKL (mixed lineage kinase domain like pseudokinase), Caspase-7 and Caspase-8 were markedly elevated in group Ⅲ as compared to the control group (*P* < 0.05) (Fig. 6**F-H**). In contrast, the protein levels of RIPK1 did not undergo a notable change (Fig. 6**E**). Similarly, immunofluorescence analysis demonstrated a notable increase in fluorescence intensity for RIPK3 (receptor-interacting protein kinase 3), MLKL, and Caspase-8 with increasing Cu concentration (Fig. 6**I-K**). The findings indicated that elevated Cu levels may induce necroptosis in chicken kidneys.

### Bioinformatics correlation analysis at the lipid metabolism and PANoptosis gene levels

**Fig. 7:**
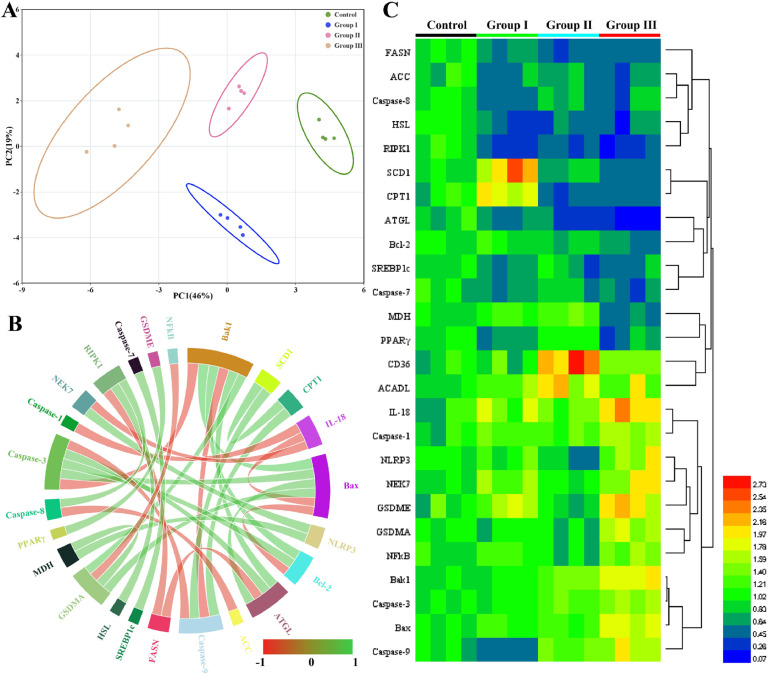
Bioinformatics correlation analysis. (A) The PCA analysis. (A) The correlation and chord plot analyses. (A) The heat map analyses.

A bioinformatics correlational analysis was undertaken to explore the association between lipid metabolism and PANoptosis. The results of the principal component analysis (PCA) demonstrated a high degree of similarity within each group and effective discrimination between the control and Cu-treated groups (Fig. 7**A**). Additionally, correlation and chord plot analyses revealed a notable correlation between lipid metabolism and PANoptosis genes (Fig. 7**B**). The heat map classification results corroborated these findings (Fig. 7**C**). The analyses indicated a correlation between lipid metabolism disorders and PANoptosis, suggesting that lipid metabolism disorders may contribute to Cu-induced PANoptosis.

## Discussion

The accelerated advancement of agriculture and industry has led to a notable increase in the cumulative impact of Cu on the environment. In consequence, the enrichment of Cu represents a significant risk to global food security. The excessive consumption of Cu has been demonstrated to elicit toxic effects in both animals and humans (Chen, et al., 2024). Furthermore, the investigation established a substantial association between Cu levels and the probability of developing CKD (Guo, et al., 2022). Considering that kidney is a target organ for Cu, it is essential to conduct a comprehensive investigation into the nephrotoxic effects of Cu. It has been demonstrated that irregularities in Cu metabolism can facilitate the pathological process of renal fibrosis (Jiayi, et al., 2024). Concurrent studies have indicated that Cu overload can result in renal mitochondrial dysfunction, cellular senescence and renal fibrosis (Zhu, et al., 2024). In the present study, excess Cu intake was found to significantly elevate serum and kidney Cu levels in broilers. Furthermore, renal and serum concentrations of iron and zinc were notably decreased in response to excess Cu intake, indicating a disruption in the balance of trace elements within the kidney. In addition, high Cu levels resulted in significant elevation of Crea and BUN, biochemical indicators of broiler kidney. HE staining showed that glomerular damage occurred, and Masson staining showed increased renal fibrosis. Meanwhile, the electron microscopy findings revealed that elevated Cu levels led to the formation of vacuoles within the mitochondria. This observation lends further support to the hypothesis that an excess of Cu can lead to renal injury.

Lipids are integral to a number of fundamental biological processes, including energy metabolism, the structure of biological membranes, signal transduction, and a range of other biological functions (Petrenko, et al., 2023). Current literature indicates that Cu regulates lipid metabolism (Zhong, et al., 2022). Exposure to iron, zinc, and Cu has been shown to promote iron overload, lipid peroxidation, and mitochondrial abnormalities in hippocampal neurons of mice (Shi, et al., 2023). As evidence that Cu affects the lipid metabolism of the Chinese mitten crab Eriocheir sinensis via the TOR-SREBP pathway (Yang, et al., 2022). Gao *et al*. (Gao, et al., 2023) demonstrated that the protective effect of *Bacillus coagulans* XY2 against Cu toxicity is primarily associated with alterations in lipid metabolism. Accordingly, disrupted lipid metabolism contributes to the development of various pathological processes. CD36 mediates the uptake of fatty acids by cells via endocytosis (Glatz, et al., 2024). The current study revealed a considerable increase in CD36 levels, thereby indicating that Cu may potentially contribute to the facilitation of fatty acid uptake by renal cells. Fatty acid synthase (FASN) plays a pivotal role in the synthesis of fatty acids, while the roles of acetyl-CoA carboxylase (ACC), stearoyl-CoA desaturase 1 (SCD1), and sterol regulatory element binding protein-1c (SREBP1c) in this process have also been demonstrated (Zhang, et al., 2023). Similarly, chronic exposure to Cu was observed to elicit a reduction in the expression of ACC, SCD1 and SREBP1c, thereby indicating that the synthesis of lipids is impaired in the kidney. The equilibrium between lipid synthesis and catabolism plays an indispensable role in maintaining lipid homeostasis. Similarly, the current investigation has revealed that lipolysis-related indices hormone-sensitive triglyceride lipase (HSL) and adipose triglyceride lipase (ATGL) were significantly diminished within the Cu cohort in chicken kidneys. The aforementioned findings indicate that an excess of Cu may result in the impairment of both lipid synthesis and catabolism in the kidney.

Fatty acid β-oxidation releases energy to sustain essential biological processes, and inhibition of this process can lead to lipid deposition. Carnitine palmitoyl transferase 1 (CPT1) plays a vital function in the transfer of fatty acids to the mitochondria. This process enables the precise regulation of fatty acid entry into the mitochondria for subsequent oxidation (Liu, et al., 2022). Meanwhile, enzymes malate dehydrogenase (MDH) and acyl-CoA dehydrogenase long (ACADL) are integral to the process of mitochondrial β-oxidation of fatty acids. Fatty acid oxidation mediated by CPT1 has been shown to be protective against peritoneal fibrosis (Su, et al., 2023). The present study indicate that excess Cu led to a significant reduction in CPT1 but promoted ACADL expression, indicating impaired fatty acid oxidation. Disorders of fatty acid metabolism a role in the development of lipophagy. Rab7 acts as a mediation of lipid droplets (LDs) replenishment by lysosomes during lipophagy, and PLIN2 is also involved in lipophagy (Piper, et al., 2024). Finally, LC3 promotes the process of lipid phagocytosis. In the present study, excess Cu induced up-regulation of Rab7, PLIN2 and LC3. The above results suggest that an excess of Cu may lead to disturbances in lipid oxidation and lipophagy, which may lead to disturbances in kidney function.

Programmed cell death (PCD) is a fundamental process in the regulation of cellular biological functions. The PANoptosome is controlled by a complex of upstream regulatory molecules and receptors, which are involved in the induction of PANoptosis (Sun, et al., 2024). The constitution of the PANoptosome, comprising Caspase-8, NLRP3, and RIPK3, results in the activation of a complex cascade of apoptosis, pyroptosis, and necrotic processes. Moreover, the inflammasome constitutes a principal component of the PANoptosome, the activation of which is of paramount importance for both cellular necrosis and PANoptosis. The activation of NLRP3 and Caspase-1 occurs intrinsically within the cell, resulting in the maturation of IL-1β and IL-18 and the acquisition of pro-inflammatory properties by focal death (Fu and Wu, 2023). Upon activation, the NLRP3 inflammasome facilitates the ASC-gasdermin D (GSDMD) pathway, which ultimately results in the death of inflammatory cells through the process of PANoptosis. The present investigation revealed that Cu exposure resulted in the activation of IL-1β and IL-18 in chick kidneys. Additionally, the concurrent overactivation of NLRP3, GSDMD and Caspase-1 in the Cu-exposed kidneys can contribute to the development of cellular pyroptosis.

Necroptosis represents a kinase-mediated variant of regulated necrosis, whereby the formation of the RIPK1-RIPK3 complex is initiated. In addition, RIPK3 can promote an inflammatory response independent of necrotic apoptosis. In renal cells, inhibition of Caspase-8 prevents apoptosis but may lead to RIPK1-mediated necrotic apoptosis (Sanz, et al., 2023). Active Caspase-8 then proceeds to initiate apoptosis through a process of cleavage, which in turn leads to the cleavage of Caspase-9 and Caspase-3. This ultimately results in the occurrence of apoptosis (Ai, et al., 2024; Gao and Zhang, 2023). The present findings demonstrate that elevated Cu levels triggered the activation of Caspase-8 and resulted in the up-regulation of Bak1, Bax, Caspase-9 and Caspase-3, while concurrently leading to a reduction in the level of Bcl-2 in the kidneys, thereby initiating the process of apoptosis. In addition, it has identified the RIPK3 protein as a pivotal regulator of necrosis, with implications in the generation of PANoptosome (Sundaram, et al., 2023). The findings of our correlation revealed a marked increase in the expression of RIPK1, RIPK3 and MLKL proteins, which together suggest that excess Cu may be involved in the development of renal necrosis. Therefore, it can be postulated that an excess of Cu may facilitate the PANoptosis by compromising mitochondrial function, thus enabling the formation of the PANoptosome.

Although we demonstrated Cu-induced disorders of renal lipid metabolism leading to biomarkers of PANoptotic signalling in *vivo* studies, the deeper signalling failed to be elucidated. Consequently, at this point it has not been possible to ascertain whether any underlying injuries are involved in activating PANoptotic signalling. Nevertheless, given that the kidney expresses all essential proteins involved in pyroptosis, apoptosis, and necroptosis, it can be postulated that PANoptosis may be the predominant mechanism involved in this process (Yuan, et al., 2024). Upon further examination, a strong correlation was found between FA metabolism and PANoptosis-related indicators. Elevated Cu levels have been demonstrated to disrupt renal lipid metabolism, potentially precipitating renal injury through initiating PANoptotic signalling in the kidney.

## Conclusion

In summary, our results demonstrate that Cu toxicity can lead to renal damage in broilers. Moreover, disturbances in lipid metabolism may contribute to Cu-induced PANoptosis, thereby exacerbating renal injury.

## Disclosures

The authors declare that they have no known competing financial interests or personal relationships that could have appeared to influence the work reported in this paper.

## Appendix Supplementary materials

Image, application 1
