# Supplementary material for: The role of lipid metabolism imbalance in copper-induced PANoptosis in broiler kidney
Source: Poult Sci. 2025 Jul 15;104(10):105549. doi: 10.1016/j.psj.2025.105549 (PMC12284046; doi:10.1016/j.psj.2025.105549)
Supplement: Supplementary file 1 [file mmc1.docx]

**Supplementary materials**

**Table S1**. qPCR primers and product size

| Gene | Gene Bank | Primer sequences (5´-3´) | PCR product size (bp) |
| --- | --- | --- | --- |
| Actin | NM_205518.2 | GAAGCCCAGAGCAAAAGAG  CTCTGTTGGCTTTGGGGTT | 181 |
| FASN | NM_205155.4 | AGGAAATGGGTATTGTCG  CAGGCTTGATACCACATT | 200 |
| ACC | XM_046929960.1 | CTGGATAACCTGGTCAACG  TCATTAGTCGCTCAACCC | 91 |
| SCD1 | NM_204890.2 | GTTTCCACAACTACCACCAT  ATCTCCAGTCCGCATTTT | 173 |
| MDH | NM_001006395.3 | AAGGTGGTAGTAGTTGGG  GTGATCCAAGCGAGTCAA | 108 |
| PPARγ | XM_046925952.1 | CCAACTCACTTATGGCTAT  CTTTTCTTATGGATGCGAC | 166 |
| CD36 | XM_046907113.1 | GGGAAAGAAGACATAAGCA  GTCAGAGGAGAAGAAGCG | 162 |
| CPT1 | XM_046918285.1 | CACAGGGCTTTGGGTTGC  TCCGTCCCGAGAAGAGTT | 149 |
| ACADL | NM_001006511.3 | ACTGACATCGGCACTCGG  CGTACATCTGCTCCTCCC | 247 |
| HSL | XM_040695201.1 | AGGAACACAACGCAGCCGAA  GCACGAACTGGAACCCGAGAC | 213 |
| ATGL | XM_046918893.1 | TCTACTGTGGGCTGATACCT  GTGGAACTGTCTCGTGGG | 151 |
| SREBP1c | NM_001001464.1 | AATCACCCAGTGGAGCAG  CTCAGACCTTGGCATTCG | 266 |
| IL-18 | XM_046932263.1 | GCAGTACGGCTTAGAGAAAA  GTACATTCCACTGCCAGATT | 198 |
| NLRP3 | XM_046918112.1 | AGCTACCACACATCTAGGAT  GGTGTCCAAATCCTCAATCT | 207 |
| Caspase-1 | XM_040687588.2 | TTCCTTCAACACCATCTACG  GGTGAGCTTCTCTGGTTTTA | 209 |
| NEK7 | XM_046922466.1 | GTATGAGATGGCTGCACTGC  CTGAAGGGAGAGGCGGATAG | 104 |
| GSDMA | XM_046933379.1 | ACCCTGAGCATTCCCATTGA  AGTTCTGCCTCACCTTCCTC | 187 |
| GSDME | XM_046911141.1 | TCTTGGCAGTCTCGTTGAGT  CTCCCAGGCTGATCTTTCCA | 150 |
| NFkB | XM_046915553.1 | TCAACGCAGGACCTAAAGACAT  GCAGATAGCCAAGTTCAGGATG | 162 |
| Bak1 | XM_046932962.1 | ATGGATGCCTGTCTGTCCTGTTC  GCAGAGCAGTCCAAAGACACTGA | 106 |
| Bax | XM_046922136.1 | TCCTCATCGCCATGCTCAT  CCTTGGTCTGGAAGCAGAAGA | 69 |
| Bcl-2 | XM_046910476.1 | GATCGTCGCCTTCTTCGAGT  GGCCTCATACTGTTGCCGTA | 217 |
| Caspase-9 | XM_046903261.1 | GGAAACCTTGGACAGCGTAC  TCTGCTTGTACCTCCCCTTG | 107 |
| Caspase-3 | XM_046915477.1 | TTGAAGCAGACAGTGGACCA -3'  GTTCAAGTTTCCTGGCGTGT -3' | 177 |
| Caspase-7 | XM_046920622.1 | GCATATTCCACAGTGCCAGG  ACCACATAGTTGACCCGTGT | 143 |
| Caspase-8 | NM_204592.4 | CGATTCTCTGGGCAACTGTG  CTGAGTTCTGGCACTGCTTC | 132 |
| RIPK1 | XM_046910230.1 | CACAGCTCTCAGGGTCAGAT  TCAGGGGTGTATGGTGGAAC | 185 |

**Table S2.** Antibody list

| Name | Information | Name | Information |
| --- | --- | --- | --- |
| β-actin | Proteintech, China | GSDMD | ABclonal, China |
| CD36 | ABclonal, China | Caspase-1 | Proteintech, China |
| CPT1 | Proteintech, China | NFkB | Proteintech, China |
| HSL | Proteintech, China | Bcl-2 | Proteintech, China |
| SREBP1 | Proteintech, China | Caspase-9 | ABclonal, China |
| RAB7 | ABclonal, China | Caspase-3 | Proteintech, China |
| PLIN2 | ABclonal, China | RIPK1 | ABclonal, China |
| LC3 | Proteintech, China | MLKL | ABclonal, China |
| IL-1 | Bioss, China | Caspase-7 | ABclonal, China |
| IL-18 | Bioss, China | Caspase-8 | ABclonal, China |
| NLRP3 | Proteintech, China |  |  |
